# A Case of Cervical and Thoracic Epidural Abscess Presenting As Diabetic Ketoacidosis Amid Persistent Bacteremia and Alcohol Withdrawal

**DOI:** 10.7759/cureus.71956

**Published:** 2024-10-20

**Authors:** Ramon Gil, Alison Thornton, Ryan Wong, Arianna Lozada, John C Romano

**Affiliations:** 1 Graduate Medical Education, HCA Florida Northwest Hospital, Margate, USA; 2 Medicine, Nova Southeastern University Dr. Kiran C. Patel College of Osteopathic Medicine, Fort Lauderdale, USA; 3 Internal Medicine, HCA Florida Northwest Hospital, Margate, USA

**Keywords:** bacteremia, diabetes, epidural, neurosurgery, staphylococcus aureus

## Abstract

This is a case of a spinal epidural abscess (SEA) in a 41-year-old male with a history of chronic alcohol abuse, who initially presented with severe diabetic ketoacidosis (DKA). Following stabilization of DKA, the patient developed persistent gram-positive bacteremia, which progressed to an epidural abscess extending from the cervical to the thoracic spine with potential intracranial extension. Despite the complexity of the case, prompt diagnosis via magnetic resonance imaging (MRI) and timely neurosurgical intervention for drainage, combined with targeted antibiotic therapy, led to a favorable outcome. SEA remains a rare but serious complication, particularly in the context of DKA and bacteremia. Early recognition and intervention are crucial to prevent neurological sequelae. This case emphasizes the importance of maintaining a high index of suspicion for SEA in patients with risk factors such as diabetes and alcohol abuse, particularly when they present with new or worsening back pain and systemic signs of infection.

## Introduction

Diabetic ketoacidosis (DKA) is a life-threatening complication of diabetes, often precipitated by infection, noncompliance or discontinuation of insulin therapy, or other stressors. It usually evolves over 24 hours. The earliest symptoms of hyperglycemia include polyuria, polydipsia, and weight loss. As the hyperglycemia worsens, lethargy, focal signs, and obtundation can occur. DKA can be complicated by severe infections, such as epidural abscesses, which further exacerbate the patient’s condition [1]. Epidural abscesses, although uncommon, are serious infections that can lead to significant morbidity and mortality if not promptly diagnosed and treated [2].

Epidural abscesses are enclosed within the confines of the spinal column. They can expand and suppress the spinal cord and cause severe symptoms. The clinical presentation can be insidious with a classic triad of fever, back pain, and neurologic deficits; however, it is uncommon to present with all three. Back pain is the most common presenting symptom. These symptoms often overlap with other conditions, making early diagnosis challenging [3]. These abscesses typically result from the hematogenous spread of bacteria, direct extension from adjacent infections, or following spinal procedures. *Staphylococcus aureus* is the most common pathogen, but other organisms, including gram-positive bacteria, can also be responsible [1]. Several conditions are associated with an increased risk of developing an epidural abscess, including diabetes mellitus, intravenous drug use, alcohol abuse, and immunosuppression [4]. The differential diagnosis for epidural abscess includes spinal malignancies, vertebral osteomyelitis, and discitis, all of which can present with similar clinical features [5]. This case report discusses a 41-year-old male with a history of alcohol abuse disorder who presented with severe DKA, complicated by persistent gram-positive bacteremia with ventral and dorsal epidural abscesses, highlighting the complex interplay of these conditions and the importance of early recognition and treatment.

## Case presentation

This is a 41-year-old male with a past medical history of alcohol use disorder and cocaine use who presented to the emergency department in South Florida with a chief complaint of shortness of breath and palpitations. While working in construction, the patient felt his heart racing and could not catch his breath. His associated symptoms included increased urinary frequency and thirst for the past few weeks. He denies burning upon urination, diaphoresis, and abdominal pain. He also had chills and fluctuating subjective temperatures of feeling cold and hot. The patient has a heavy history of alcohol abuse and reports drinking approximately eight beers per day for the past 10 years. His last drink was two days before admission. The patient is a nonsmoker and has a history of cocaine usage but no intravenous (IV) drug usage. The patient has had no prior diagnosis of diabetes. Further, the patient is uninsured.

In the emergency department, the patient’s vitals were the following: temperature, 98.2°F; blood pressure, 166/90 mmHg; heart rate, 133 bpm; and respiration rate, 18. On physical examination, the patient had Kussmaul respirations. The patient’s arterial blood gas (ABG) values were the following: pH, 7.19 (7.35-7.45); pCO_2_, 10 (35-45 mmHg); pO_2_, 122 (80-95 mmHg), HCO3, 4 (22-28 mmol/L), and other pertinent lab work consisting of serum chemistry, complete blood count, and urinalysis are presented in Table 1. 

**Table 1:** Patient’s laboratory workup on admission

Test name	Lab value	Reference range
Sodium	126	135-145 mmol/L
Potassium	4.4	3.5-5.2 mmol/L
Glucose	383	70-11 mg/dL
AST	75	10-40 U/L
ALT	74	10-60 U/L
Alkaline phosphatase	192	20-120 U/L
Total protein	9.2	5.5-8.7 g/dL
Albumin	4.6	3.2-5 g/dL
Magnesium	2.4	1.6-2.4 mg/dL
Lactic acid	2.4	0.4-2.0 mmol/L
White blood cell	13.6	4.0-10.5 x 10^3^/uL
Absolute neutrophils	11.71	1.56-6.13 x 10^3^/uL
Monocytes #	1.34	0.3-0.82 x 10^3^/uL
Lymphocytes #	0.3	1.32-3.57 x 10^3^/uL
Urine protein	100 (2+)	Neg-trace mg/dL
Urine ketones	>150 (4+)	Neg-trace mg/dL
Urine glucose	>1000 (4+)	Norm-trace mg/dL

Ultimately, the patient was found to be in a severe DKA and leukocytosis. He was admitted to the intensive care unit for immediate insulin drip and electrolyte management. Additionally, the patient was given thiamine, folic acid, lorazepam 4 mg, and chlordiazepoxide 50 mg for alcohol withdrawal.

Clinical course

On the second day of admission, the patient no longer complained of shortness of breath; however, he reported a mild headache. He was also slow to respond. The patient's vital signs remained stable and afebrile. Blood cultures were positive for methicillin-sensitive *Staphylococcus aureus* and vancomycin 1.25 g was started. Over the next two days, the patient was in alcohol withdrawal and developed mild confusion, drowsiness, and dizziness secondary to chlordiazepoxide without headaches. His DKA had resolved. Lipase values were 767 U/L (reference range 23-300 U/L). Questionable inflammatory changes surrounding the proximal duodenum in the pancreatic head region were observed on computed tomography scan. Duodenitis secondary to alcohol abuse was suspected, and the patient was started on pantoprazole 40 mg daily. A 2D echocardiogram showed a normal ejection fraction (55%-60%). Blood cultures continued to be positive for *Staphylococcus aureus*. Vancomycin was discontinued and switched to piperacillin/tazobactam. 

**Figure 1:**
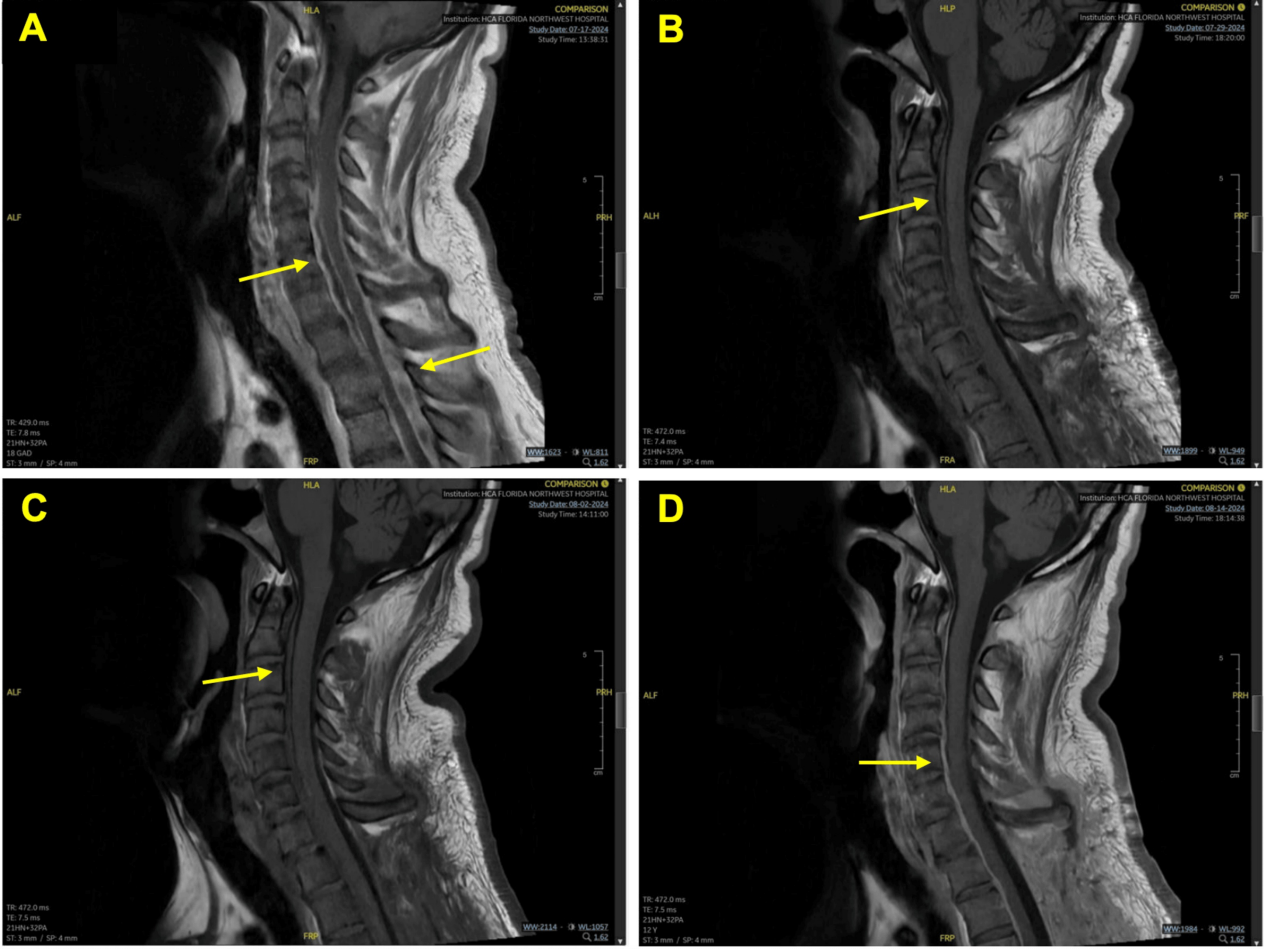
Cervical magnetic resonance imaging (MRI) (A) Initial cervical MRI revealing ventral C2-T1 spinal epidural abscess and dorsal T1-T4 spinal epidural abscess. (B) Post T1-T3 laminectomy MRI of the cervical spine. (C and D) Interim period MRI demonstrating persistent ventral spinal epidural abscess

**Figure 2:**
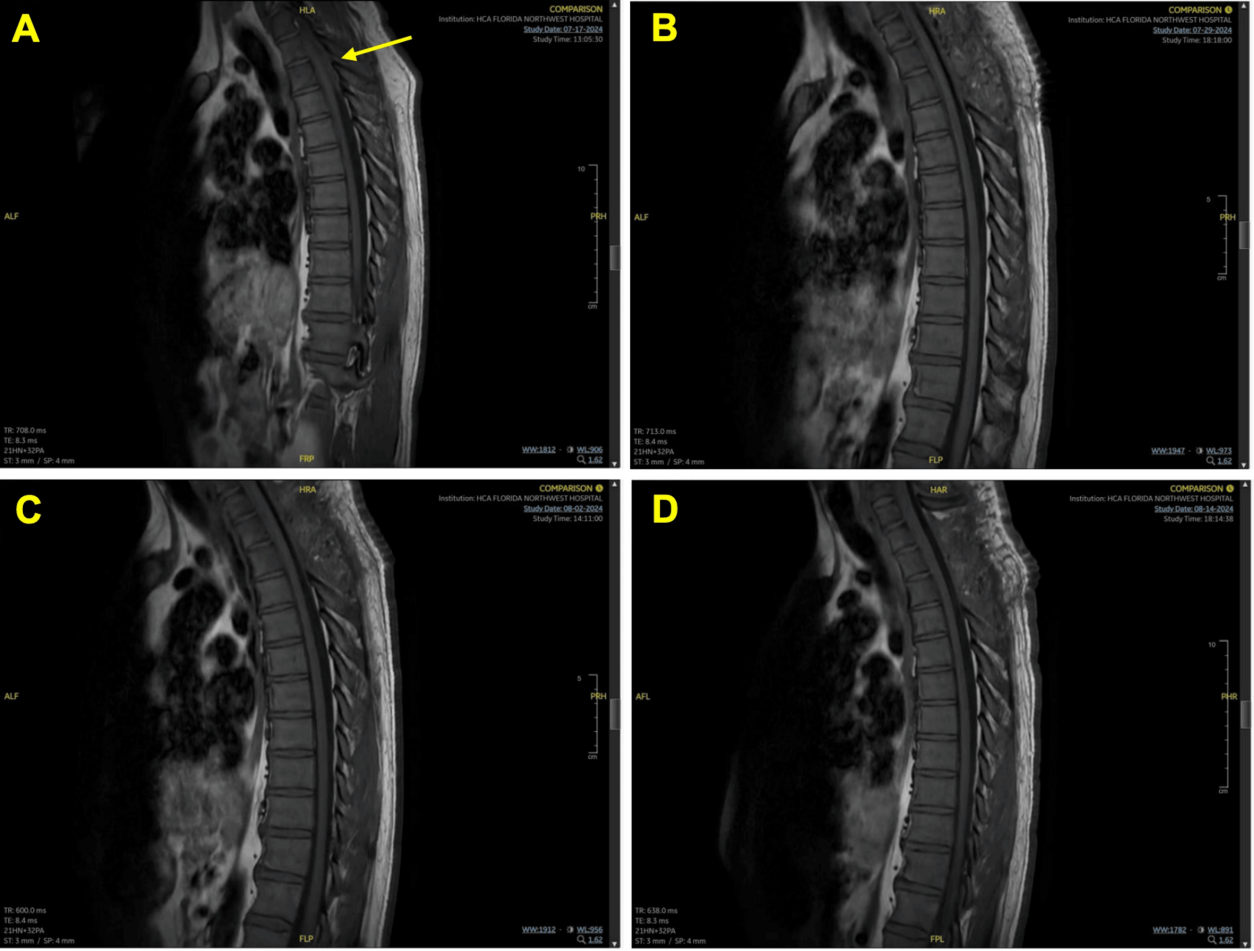
Thoracic magnetic resonance imaging (MRI) (A) Initial thoracic MRI diagnosis revealing dorsal T1-T4 spinal epidural abscess. (B-D) Post T1-T3 laminectomy MRI of the thoracic spine and interim period MRI demonstrating resolved dorsal spinal epidural abscess

Five days after admission, chlordiazepoxide was discontinued and as needed lorazepam dosing according to the Clinical Institute Withdrawal Assessment protocol was in place. The patient was more responsive; however, he developed sore pain in the upper extremities bilaterally and was febrile (101.5°F). The transesophageal echocardiogram did not reveal infective endocarditis. Three days later, blood cultures showed persistent *Staphylococcus aureus*, and piperacillin/tazobactam was increased to 4.5 g. The patient complained of minor upper back pain. On palpation, there was spinal tenderness. Magnetic resonance imaging (MRI) brain was done and demonstrated a ventral epidural abscess extending from the level of C2-T1 with possible intracranial extension (Figure 1A). The dorsal epidural collection was also observed from the level of T1-T4 (Figure 2A). There was also moderate thecal sac compression. Piperacillin/tazobactam was discontinued and switched to cefazolin 2 g. Eleven days after admission, blood cultures showed no growth.

On the next day, the patient underwent T1-T3 laminectomy due to the abscess dorsal to the spinal cord for source control. Intraoperatively, there was an abundance of granulomatous thickened tissue that was adherent to the dura. The infectious tissue was vascularized and bloody, without evidence of liquid pus. Phlegmon tissue from the dura was removed, and the cavity was irrigated. Upon wound closure, vancomycin powder and a Hemovac drain were placed. Surgical cultures were positive for *Staphylococcus aureus*. 

Postoperatively, IV cefazolin was continued, and a repeat MRI of the cervical spine revealed a persistent but resolving epidural abscess (Figures 1B-1C). No radiographic evidence of prior dorsal epidural abscess in the thoracic region was observed on MRI (Figures 2B-2C). On postop day 14, the patient developed neutropenia with a white blood cell count of 3.8 x 10^9^/L. Subsequent white blood cell count one week later decreased to 1.8 x 10^9^/L secondary to cefazolin. Antibiotic coverage was switched to nafcillin 2 g and resolved the patient’s neutropenia. Repeat MRI of the cervical spine demonstrated mild interval improvement with ventral epidural enhancement measuring a maximum transverse diameter of 1.8 mm at the level of C6 (Figure 1D). In contrast, the thoracic spine was negative for enhancement and was unchanged from prior imaging (Figure 2D). The patient was discharged on postop day 25 on IV nafcillin three times a week for six weeks without any neurological deficits.

## Discussion

This case report emphasizes the importance of a diagnostic workup of spinal epidural abscess (SEA) in a patient with a history of chronic alcohol abuse who presents with severe DKA. In this patient, the initial presentation of DKA was further complicated by concurrent alcohol withdrawal and persistent gram-positive bacteremia leading to an epidural abscess extending from the cervical to the thoracic spine. Such clinical presentation proved to be a significant factor in the somewhat delayed diagnosis and treatment. The time to diagnosis was eight days, while the time to surgical intervention was 11 days. Despite the challenges faced, prompt diagnosis via MRI, timely neurosurgical intervention for drainage, and targeted antibiotic therapy, a favorable outcome resulted. This highlights the significance of early recognition and intervention in cases of SEA, especially in high-risk patients with diabetes and alcohol abuse [6].

Several cases in the literature have reported similar presentations in which patients have SEA in the setting of DKA. One patient presented with altered mental status and undiagnosed diabetes with a pan-SEA [7]. Another patient with a known diabetes diagnosis presented with acute bilateral pan-thoracic pain and had an abscess from T4 to L1 [8]. Our patient had a segment of cervical abscess, which has been reported to occur at a lower incidence compared to thoracic and lumbar [9]. The ventral nature of his cervical SEA made it difficult to manage surgically; however, there is a lower incidence of neurological damage and morbidity reported in the literature when compared to a circumferential presentation [9]. What distinguishes our patient from these cases is the presentation of initial symptoms, in which back pain and neurological symptoms were absent. Further, the delayed presentation of neurological or musculoskeletal symptoms in this patient was likely masked due to the patient’s symptoms of alcohol withdrawal, i.e., drowsiness and lethargy from chlordiazepoxide. Hence, the management of infectious etiologies becomes complicated amid alcohol withdrawal. 

The patient's heavy use of alcohol may have influenced the extent of the bacteremia experienced. A study found that higher levels of alcohol correlated with a high level of bacteria DNA expression in serum. Endotoxins from gram-negative organisms increased in serum in 30 minutes and persisted at high levels for three hours after ingestion. Alcohol is also known to increase the permeability of the intestine, leading to bacteria translocation [10]. Alcohol is also an effective analgesic that can increase pain threshold and reduce pain intensity. If the patient did have any symptoms of SEA, it would be difficult to assess given his alcohol abuse history [11].

The other notable nature of our patient’s case was persistent bacteremia. The patient had positive blood cultures on four occasions over seven days. Interestingly, a study found that patients with concomitant infections increased the occurrence of a delayed diagnosis and obscured the symptoms of SEA [12]. The SEA in our patient may have developed due to hematogenous spread; however, the inciting cause of his bacteremia remains unknown. This does not suggest that an MRI is warranted in a patient with bacteremia and DKA, as imaging is indicated when highly suspicious symptoms originate from organs or soft tissue structures. Further, the patient's clinical status and length of time required for the exam hindered earlier diagnosis. Regarding antibiotic treatment, it has been reported that SEA accompanied by bacteremia requires more than eight weeks [12]. The patient presented herein received IV antibiotics for a total of 78 days, longer than what is conventionally advised, but was appropriate given the extensive nature of his SEA postoperatively.

## Conclusions

Overall, the implications of this case report extend to clinical practice by emphasizing the importance of a comprehensive evaluation and history taking in patients with DKA and risk factors for SEA. It reinforces the need for early imaging studies, such as MRI, and timely intervention to prevent adverse neurological sequelae and improve patient outcomes. This case contributes to the existing literature, which demonstrates an incidence increase of SEA, by providing valuable insights into the management of SEA in the context of DKA and bacteremia. Although rare, it is important to keep SEA high as a differential diagnosis when patients present with DKA symptoms.
